# Ulcerative Proctitis: An Unusual Case of Intestinal Spirochetosis

**DOI:** 10.7759/cureus.33046

**Published:** 2022-12-28

**Authors:** Jeffrey Baum, Jung Won, Moshe Fenster, Joseph Correa, Raghav Bansal

**Affiliations:** 1 Surgery, Mount Sinai South Nassau, Oceanside, USA; 2 Internal Medicine, Wyckoff Heights Medical Center, Brooklyn, USA; 3 Internal Medicine, Mount Sinai South Nassau, Oceanside, USA; 4 General Surgery, New York Medical College, Westchester Medical Center, Westchester, USA; 5 Gastroenterology, Wyckoff Heights Medical Center, Wyckoff, USA

**Keywords:** non-treponemal spirochetes, colonic ulcerations, ulcerative proctitis, human immunodeficiency virus, intestinal spirochetosis

## Abstract

Human intestinal spirochetosis (HIS) is an uncommon disease characterized by the colonization of spirochetes in the colorectal mucosa and is most often found in individuals who are positive for human immunodeficiency virus (HIV) and in homosexual men. Although HIV is known to cause a variety of infectious colitis, the prevalence has significantly declined with antiretroviral therapy. Intestinal spirochetosis, however, remains meaningful as it can be an infectious cause of colonic ulcerations even with well-controlled HIV. Spirochetosis rarely causes macroscopic changes in the colorectal mucosa and reports of an ulcerated rectum are exceedingly scarce. Here, we report a case of a homosexual man with HIV who is compliant with antiretroviral therapy with high CD4 counts who presented with a six-week history of bloody diarrhea and was found to have multiple ulcerations in the rectosigmoid junction and rectum infected with non-treponemal spirochetes as confirmed on biopsy. To our knowledge, there have not been any reports of multiple rectal ulcerations caused by non-treponemal spirochetes. The patient was treated with metronidazole 500 mg four times daily for 10 days with complete resolution of symptoms. This case is notable as it alerts clinicians to consider intestinal spirochetosis as a differential diagnosis in the workup for bloody stool in the presence of colorectal ulcerations.

## Introduction

Intestinal spirochetosis (IS) is caused by a group of bacteria that are morphologically distinct from other bacteria on account of their spiral shape. Infection with spirochetes is exceedingly rare and is most commonly found in underdeveloped countries, in men with human immunodeficiency virus (HIV) and in men who have sex with men [1]. Spirochetosis rarely causes macroscopic changes in the colorectal mucosa and reports of an ulcerated rectum are markedly rare. Clinical presentation ranges from asymptomatic to watery or bloody diarrhea to fatally invasive spirochetemia. Here, we present a rare case of a homosexual male with HIV found to have symptomatic IS with multiple rectal ulcerations.

## Case presentation

**Figure 1:**
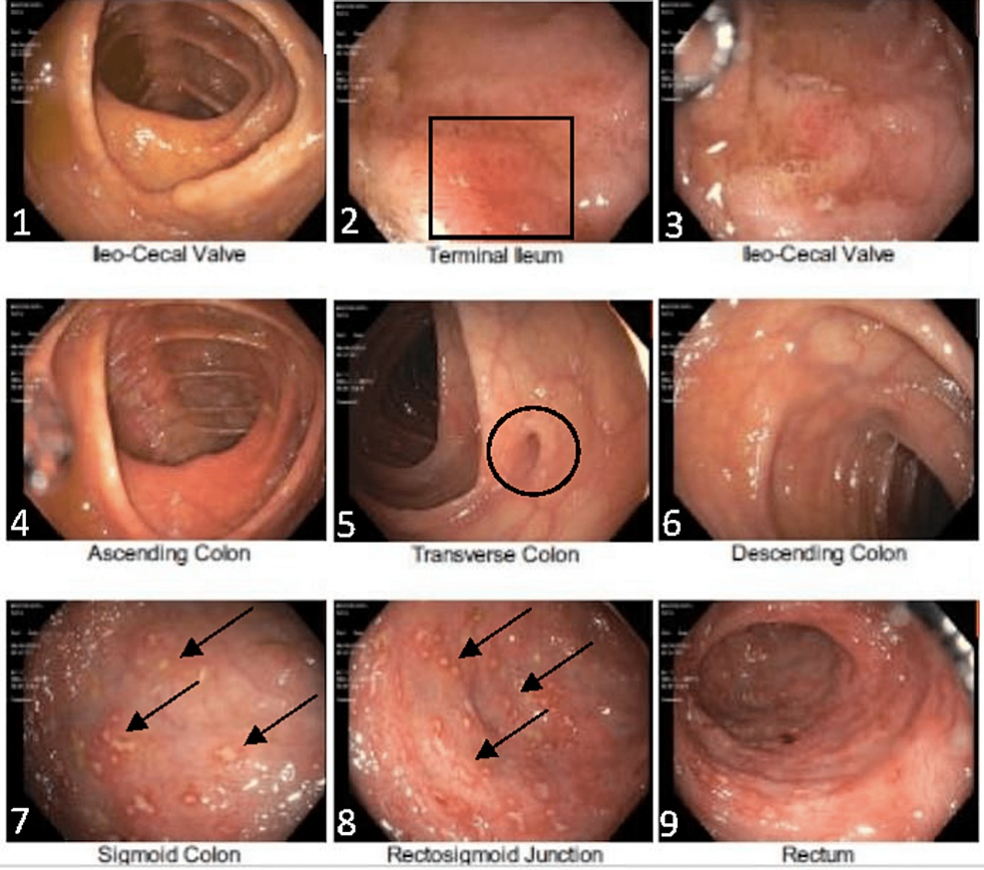
Diverticulosis without perforation or abscess is seen in the transverse colon (black circle); a single erosion is visualized in the terminal ileum (black square) and multiple ulcerations are located in the sigmoid junction and rectum (black arrows).

A 33-year-old male patient with a past medical history of HIV controlled with Biktarvy presented to our clinic complaining of six weeks of progressively worsening rectal pain with bleeding, most severe while passing stool. He described daily normal-colored stool with associated straining and bright red blood in the toilet bowl. He denied any nausea, vomiting, abdominal pain, constipation, diarrhea, recent illness, fever or sick contacts. A review of other symptoms was negative and his social history was significant for sexual activity with men. On physical exam, he was afebrile with normal vital signs. His abdomen was soft, non-tender and non-distended with normal bowel sounds. There was significant tenderness on the rectal examination. Both internal and external hemorrhoids were seen without any active bleeding, for which he took Colace 100 mg per day and Anusol Hydrocortisone 2.5% rectal cream. The rest of the physical examination findings were within normal limits. His laboratory values (Table 1) were significant for HSV 1 and HSV 2 antibodies, as well as elevated sedimentation rate and C-reactive protein. An ELISA for Entamoeba Histolytica IgG was negative. Cytomegalovirus IgM and IgG were negative. Testing for *Treponemal pallidum* spirochete was negative. All other laboratory values were within normal limits. On colonoscopy exam, diverticulosis without perforation or abscess was seen in the transverse colon; a single erosion was found in the terminal ileum and multiple ulcerations were seen in the rectum (Figure 1).

**Table 1:** Laboratory values with reference ranges for herpes simplex virus 1 and 2 (HSV 1 & HSV 2 antibodies), erythrocyte sedimentation rate (ESR) and C-reactive protein (CRP).

Lab	Unit	Patient’s Value	Reference Range
HSV1	IgG	+	-
HSV2	IgG	+	-
ESR	mm/hr	110	0-22
CRP	mg/L	26.0	0.20-3.00

 

**Figure 2:**
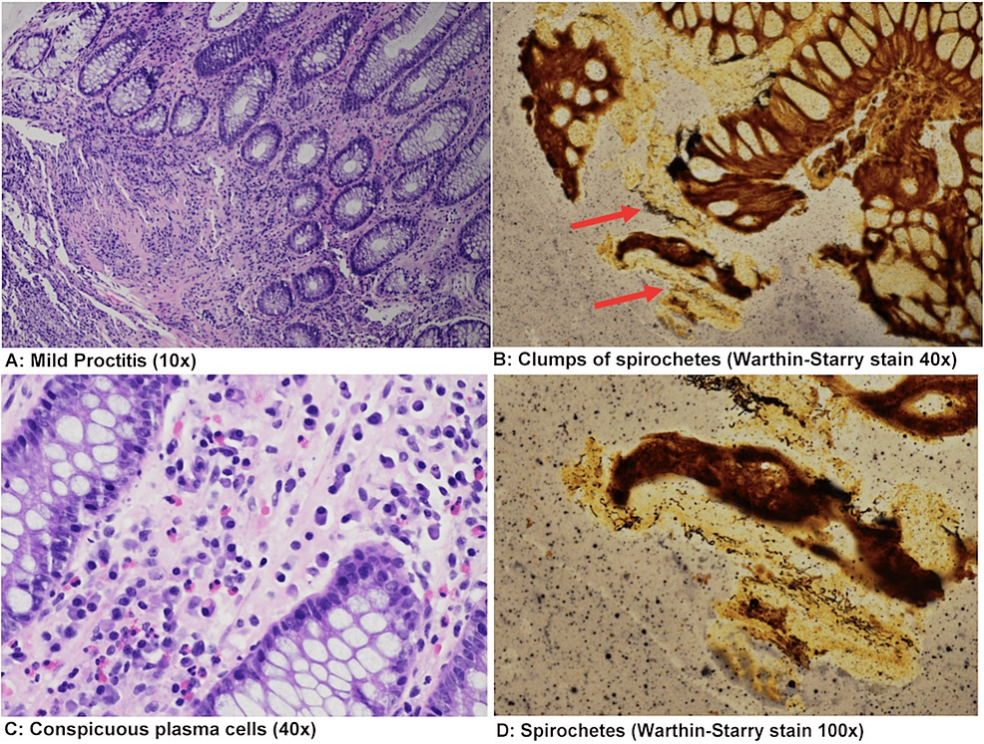
Histological preparation of rectal biopsy capturing mild proctitis at 10x magnification (A); clumps of spirochetes seen on Warthin-Starry stain at 40x magnification (red arrows, B); conspicuous plasma cells at 40x (C); Clumps of spirochetes seen on Warthin-Starry stain at 100x magnification (D).

Biopsy from the ileum and transverse colon showed no pathological alteration. Rectal biopsy revealed moderately active colitis with spirochetes identified on Warthin- Starry and immunohistochemical stain for spirochetes (Figures 2A-2D).

No viral inclusions were identified on cytomegalovirus testing. The diagnosis of ulcerative proctitis with spirochetosis was preliminarily made based on the exclusion of other causes. The patient was treated with Metronidazole 500 mg four times per day for ten days and symptoms resolved with no recurrence.

## Discussion

In humans, intestinal spirochetosis is caused predominately by the slowly growing fastidious anaerobes *Brachyspira aalborgi* and *Brachyspira pilosicoli *[2]. The exact pathogenicity of IS remains to be elucidated owing to its largely asymptomatic presentation, however, some investigators have attributed the clinical significance to the well-described spirochetes’ colonization and formation of a “false brush border” on colonic epithelium [3].

The true prevalence of IS is difficult to determine and appears to be globally dependent. Studies have reported the incidence rates in developed countries to be from 1.1% to 5% [4,5], while higher incidences are seen in individuals infected with HIV; rates in homosexual males in the United States are as high as 20% to 60% [6,7]. For this reason, it has been hypothesized to classify intestinal spirochetosis as a sexually transmitted disease [8].

IS is mostly an incidental finding on colonic biopsy with a typically asymptomatic clinical presentation [6]. The symptomatic disease process runs the spectrum from mild, watery diarrhea with or without abdominal pain to invasive spirochetemia, with a handful of case reports describing fatalities [1,9]. Interestingly, although higher rates of symptomatic human IS colonization are seen in HIV-infected individuals, there seems to be no correlation between the degree of immunodeficiency and disease severity [10]. Our patient presented with multiple bouts of bloody diarrhea and rectal pain and numerous colorectal ulcerations were visualized on colonoscopy. His past medical and sexual history, in combination with the presence of spirochetes as confirmed on biopsy, made IS the most probable diagnosis. Treatment of symptomatic IS is generally with Metronidazole 500 mg four times daily for 10 days [11,12]. Our patient was treated accordingly, with complete resolution of symptoms and no recurrence to date.

Our case marks a rare, important presentation of IS. Previous findings have described the morphology of the colonic mucosa in the presence of these organisms as “normal appearing mucosa” with a minority of cases reporting a “polypoid” appearance, one case noted an “erythematous” mucosa, and one documentation of a single questionable “lesion” [13]. Indeed, in a large 12-year study, only 17 patients were found to have IS, all of which showed only microscopic colitis [12]. The handful of case reports of multiple colorectal ulcerations has been found to be caused by either the syphilitic spirochete* Treponema pallidum* [14,15] or concomitantly in the erosive inflammation associated with ulcerative colitis [16]. Our patient, however, had no previous diagnosis of UC, and had a negative Treponemal test, yet presented with multiple colorectal ulcerations found to be infected with spirochetes. To our knowledge, there are no reports of multiple rectal ulcerations caused by non-treponemal spirochetes in a patient without a previous diagnosis of ulcerative colitis.

## Conclusions

In conclusion, this case report demonstrates evidence of HIS causing symptomatic bloody diarrhea along with multiple rectal ulcerations in a homosexual male with HIV. The ruling out of common causes of colorectal ulcerations should alert clinicians to consider spirochetosis as a possible causal agent and swift administration of appropriate antimicrobials should be initiated.
